# Physical exercise and depression in college students: the serial mediating roles of mindfulness and experiential avoidance

**DOI:** 10.3389/fpsyg.2026.1838963

**Published:** 2026-06-22

**Authors:** Zhihua Zheng, Wei Xu, Jianhua Shen, Weijun Yu, Wenzeng Xu

**Affiliations:** 1 Affiliated Dinghui Experimental Primary School of Zhejiang Normal University, Hangzhou, China; 2 School of Physical Education and Health, Yancheng Teachers University, Yancheng, China; 3 Jing Hengyi School of Education, Hangzhou Normal University, Hangzhou, China; 4 Faculty of Sport, College of Teacher Education, Taizhou University, Taizhou, China; 5 College of Medicine, Zhejiang University, Hangzhou, China

**Keywords:** college students, depression, experiential avoidance, mindfulness, physical exercise, serial mediation

## Abstract

**Background:**

Guided by Acceptance and Commitment Therapy (ACT), this study examined whether trait mindfulness and psychological inflexibility and experiential avoidance-related processes, assessed using the Acceptance and Action Questionnaire-II (AAQ-II), were statistically linked to the association between physical exercise and depressive symptoms among college students.

**Methods:**

A cross-sectional cluster sample was recruited from 11 general physical education classes at one university in Jiangsu Province, China. A total of 308 valid questionnaires were retained from 320 invited students. Participants completed the Physical Activity Rating Scale-3 (PARS-3), Mindful Attention Awareness Scale (MAAS), Acceptance and Action Questionnaire-II (AAQ-II), and Self-Rating Depression Scale (SDS). Data were rechecked for missing values, duplicate records, out-of-range values, and potential outliers. Welchupl*t*-tests, Pearson correlations, standardized regression path models, and bootstrap-based serial indirect association analyses were conducted in R. Gender was included as a covariate.

**Results:**

The prevalence of at least mild depressive symptoms was 20.78% based on SDS standard scores. Physical exercise was negatively associated with depressive symptoms (β = −0.264, *p* < 0.001) and positively associated with mindfulness (β = 0.286, *p* < 0.001). After mindfulness was included, the direct association between physical exercise and AAQ-II-related scores was not significant (β = −0.083, *p* = 0.113). Mindfulness was negatively associated with AAQ-II-related scores and depressive symptoms, whereas AAQ-II-related scores were positively associated with depressive symptoms. Bootstrap analyses showed a significant indirect association through mindfulness and a significant serial indirect association through mindfulness followed by AAQ-II-related scores. The indirect path through AAQ-II-related scores alone was not significant.

**Conclusion:**

Physical exercise was associated with lower depressive symptoms among college students, and this association was statistically linked to trait mindfulness and AAQ-II-related scores in a serial pattern. AAQ-II-related scores did not operate as an independent indirect pathway but appeared mainly within the pathway following mindfulness. Because the study was cross-sectional and based on self-report data from a single university, the findings should be interpreted as cross-sectional indirect associations rather than evidence of causal mediation.

## 1 Introduction

Depression refers to a cluster of mood-related manifestations, including persistent low mood, anhedonia, and fatigue, which are closely associated with individuals’ cognitive appraisal of stressful events, coping resources, and social support (Jesulola et al., 2018). The college stage represents a critical developmental period in the transition from adolescence to young adulthood, during which individuals face multiple pressures including academic demands, interpersonal relationships, and self-identity issues. Consequently, common mental health problems, such as depression, are highly prevalent in this population (Casey et al., 2022). A longitudinal survey of Chinese college students reported that 20–40% of undergraduates have experienced varying degrees of depression, anxiety, and stress, with approximately 35% exhibiting depression levels higher than those in the general population (Liu et al., 2019). Although subclinical depression in college students does not meet clinical diagnostic criteria, its long-term presence may still impair learning efficiency and quality of life. These symptoms can also negatively affect social adaptation and development (Jamil et al., 2022). Given the high prevalence of depression among college students and its far-reaching impact, it is necessary to explore contributing factors in depth and develop effective prevention and intervention strategies. Existing research suggests that physical exercise is associated with lower depressive symptoms and may serve as an important protective correlate of mental health (Heissel et al., 2023). Regular participation in physical exercise has been linked to lower depressive symptoms among college students, and intervention studies suggest that exercise may be beneficial for individuals with mild to moderate depressive symptoms. This broader evidence base is supported by meta-analytic findings showing that exercise is associated with reduced depressive symptoms, even after adjusting for publication bias (Schuch et al., 2016). However, evidence from exercise randomized controlled trials should also be interpreted cautiously, as control group responses may influence estimates of antidepressant effects in exercise trials (Stubbs et al., 2016). Although the preventive and ameliorative effects of physical exercise on depression have been confirmed by many studies (Rezaie et al., 2023; Chen et al., 2024), the academic community has not yet reached a consensus on the modality, intensity, frequency, and duration of exercise interventions. Mechanism-focused reviews further suggest that physical activity may be linked to youth mental health through neurobiological, psychosocial, and behavioral pathways, although these pathways remain heterogeneous across populations and activity contexts (Lubans et al., 2016). Quantifiable standards for personalized interventions are also lacking (Browning et al., 2019). In this context, integrated intervention models that combine psychotherapeutic approaches have gradually attracted attention. Acceptance and Commitment Therapy (ACT) goes beyond the traditional concepts of cognitive behavioral therapy and offers a new perspective on the causes of depression, and has achieved good empirical results in addressing symptoms such as depression among college students (Gloster et al., 2020; Knight and Samuel, 2022). This theoretical perspective provides a new research direction for exploring potential psychological pathways linking physical exercise with depressive symptoms among college students.

### 1.1 Acceptance and commitment therapy

ACT is a third-wave cognitive behavioral therapy proposed in the 1990s by American psychologist Steven C. Hayes and others, based on functional contextualism and Relational Frame Theory. In contrast to traditional cognitive behavioral therapy (CBT), which aims to modify the content of one’s thoughts, ACT focuses more on the circumstances surrounding an individual’s thoughts and actions (Greco et al., 2008). In this way, individuals can respond more flexibly to their thoughts, emotions, and bodily sensations, and pursue a full and meaningful life (Schumacher et al., 2025). Psychological flexibility is the hallmark of ACT and its most important treatment outcome (Hayes et al., 2006). These processes collectively enhance psychological flexibility through strategies such as cognitive defusion mindfulness practice, values clarification, and acceptance (Hayes et al., 1999). Because of its treatment philosophy centered on enhancing psychological flexibility and its flexible and practical intervention model, ACT has been widely applied in multiple fields such as psychology and medicine. Research shows that ACT can effectively improve emotional problems such as anxiety (Swain et al., 2013) and depression (Fang and Ding, 2020). It also demonstrates significant effects and practical value in enhancing individuals’ social adaptation (Corti et al., 2018).

### 1.2 Related research on physical exercise, mindfulness, and depression

In recent years, the protective effect of physical exercise on depression has attracted increasing academic attention. Related studies have systematically examined the intervention effect of physical exercise on depression, focusing on different types of exercise, intensity, frequency, and duration (Chen et al., 2024; Zhang et al., 2025). Existing studies have indicated that regular physical exercise plays a positive role in alleviating depression, with a significant intervention effect on mild to moderate depression among college students (Zhang et al., 2022). Mindfulness, as a core foundation and important component of ACT, is both a psychological trait and a practice (Kabat-Zinn, 2003). Studies have found that mindfulness can serve as an important protective factor, buffering the adverse impact of negative mental states on mental health (Li et al., 2025), and reducing the likelihood of depression (Gál et al., 2021; Tan et al., 2022). With further research, the close association between physical exercise and mindfulness levels has gradually emerged. Physical exercise can guide individuals to focus attention on present-moment bodily sensations, while effectively weakening negative cognitive rumination (Liu et al., 2023). Forms of physical exercise that emphasize bodily sensations, such as yoga and aerobic exercise, help improve individuals’ mindfulness levels (Mothes et al., 2014; Cox and McMahon, 2019). Evidence from meditative movement interventions, including practices that combine bodily movement, attention regulation, and mindful awareness, also suggests potential relevance for depressive symptoms (Zou et al., 2018; Zhu et al., 2024). The above studies provide theoretical and empirical support for physical exercise positively predicting mindfulness levels. In summary, physical exercise may be associated with lower depressive symptoms, and mindfulness may be an important psychological factor linking physical exercise with depression. The existing literature provides theoretical and empirical support for examining mindfulness as a potential indirect pathway in this association. Accordingly, this study proposes the following hypothesis:

*H1:* Mindfulness will be statistically associated with an indirect pathway linking physical exercise and depressive symptoms.

### 1.3 Research on the relationships among physical exercise, experiential avoidance, and depression

Experiential avoidance is a core concept of ACT. It refers to rigid, long-term behavioral and cognitive strategies that individuals adopt to avoid, control, or alter unwanted internal experiences (Mugarura et al., 2025). As a vulnerable psychological manifestation, experiential avoidance is an important predictor of depression (Torunsky et al., 2023). ACT posits that the key to alleviating depression is not to change the content of cognition itself, but to change an individual’s relationship with thoughts and emotions. It guides individuals to view internal experiences with acceptance and openness, and to take actions consistent with their values (Hayes et al., 2012). Psychological flexibility refers to the ability to be fully engaged in the present, to refrain from unnecessary resistance or avoidance when facing negative thoughts and emotions, and to take actions consistent with one’s values. Psychological flexibility and experiential avoidance are conceptually opposed psychological processes. Experiential avoidance is highly correlated with the occurrence of depression (Chawla and Ostafin, 2007; Mellick et al., 2019). Therefore, enhancing psychological flexibility and reducing experiential avoidance are important mechanisms for promoting individual mental health, and are important pathways for alleviating depression among college students.

There is academic consensus on the preventive and intervention effects of physical exercise on depression. However, whether physical exercise is associated with lower experiential avoidanced intervention effects of physicato depressionted with lower experiential avoidanced interventioThe psychological and behavioral characteristics inherent in physical exercise provide important potential for intervening in experiential avoidance. ACT points out that the excessive dominance of language and cognition is an important premise leading individuals to develop experiential avoidance (Bond et al., 2011). Physical exercise centers on bodily experience and action-oriented engagement (Xu, 2020). Its process emphasizes bodily perception, which may weaken the excessive dominance of verbal cognition over behavior and may therefore be associated with lower levels of experiential avoidance. Second, experiential avoidance causes individuals to focus excessively on controlling negative psychological states, leading them to detach from the real situation of the present and to lack clear awareness of current behavior (Berzonsky and Kinney, 2019). Physical exercise requires individuals to focus on the bodily experience of the present. Regular physical exercise can enhance individuals’ ability to perceive the present. This view has been preliminarily verified in related research. For example, some scholars explored the effects of mindfulness yoga on improving depression and anxiety through an intervention combining mindfulness and yoga (Yang et al., 2023), providing empirical support for the above logical deduction.

The effect of physical exercise on experiential avoidance may not be singular. ACT theory points out that experiential avoidance arises from the rigid control of internal experiences by the individual, and mindfulness is a key pathway to improve this relationship. This means that the effect of physical exercise on experiential avoidance may be partially mediated by increasing mindfulness levels, rather than occurring independently. Therefore, it can be inferred that the effect of physical exercise on experiential avoidance may be complex. It is necessary to include mindfulness in the model and examine the synergistic effect of mindfulness and experiential avoidance in the chain pathway. The above analysis shows that physical exercise and experiential avoidance have opposite characteristics in psychological processes. Accordingly, it can be inferred that physical exercise has a negative predictive effect on experiential avoidance. Based on the theoretical links and empirical evidence among the three factors of physical exercise, experiential avoidance, and depression, this study proposes the following hypothesis:

*H2*: Experiential avoidance mediates the effect of physical exercise on depression.

### 1.4 Related research on the relationships among physical exercise, mindfulness, experiential avoidance, and depression

Research has shown that mindfulness can reduce individuals’ tendency toward experiential avoidance through three unique psychological processes. First, conscious awareness of the present allows individuals to face internal experiences that they want to avoid. Second, a nonjudgmental attitude means that individuals do not deny or reject negative experiences. Third, a decentered cognitive mode creates distance between the self and negative psychological events, reduces the sense of threat of negative experiences, and thus renders individuals more willing to accept rather than avoid (Moran et al., 2021). Since ACT proposed the concept of experiential avoidance, many studies have confirmed that mindfulness, as an important psychological trait, can effectively enhance individuals’ psychological tolerance and level of acceptance (Baer et al., 2006). It is an important pathway for reducing experiential avoidance (Zhang et al., 2021). The above analysis shows that mindfulness and experiential avoidance are closely related to physical exercise and depression. They are also closely related to each other. Based on this, this study proposes the following hypothesis:

*H3*: Mindfulness negatively predicts experiential avoidance, and mindfulness-experiential avoidance plays a serial mediating role between physical exercise and depression.

In summary, based on the ACT theory regarding the causes of depression, this study draws on two core psychological factors of ACT—mindfulness and experiential avoidance—to construct a serial mediation model. The model aims to explain mindfulness and experiential avoidance in terms of how physical exercise alleviates depression and to provide empirical evidence for improving the intervention effect of physical exercise on the mental health of college students.

## 2 Materials and methods

### 2.1 Study design and participants

This study used a cross-sectional design. Participants were recruited by cluster sampling from 11 general physical education classes at a university in Jiangsu Province, China. A total of 320 students were invited to complete the questionnaire. After invalid responses were excluded, 308 valid questionnaires were retained, yielding a valid response rate of 96.25%. The final sample included 198 women (64.29%) and 110 men (35.71%). Because the sample was drawn from a single university in Jiangsu Province and included a higher proportion of women than men, it should not be considered nationally representative of the overall population of Chinese college students. Sex was included as a covariate in subsequent analyses to reduce the influence of the unbalanced sex distribution on the interpretation of the results; however, this statistical adjustment cannot fully address the limitations in external validity arising from the single-site sample and sex imbalance.

### 2.2 Measures

#### 2.2.1 Physical exercise

Physical exercise was measured using the Physical Activity Rating Scale-3 (PARS-3). The scale was originally developed by Hashimoto (1990) and later revised in Chinese by Liang (1994). It assesses the intensity, duration, and frequency of physical exercise. Exercise volume was calculated as follows: Exercise volume = intensity × (duration - 1) × frequency. The total exercise volume score ranges from 0 to 100. According to established criteria, scores ≤ 19 indicate low exercise volume, scores of 20–42 indicate moderate exercise volume, and scores ≥ 43 indicate high exercise volume.

Because trait mindfulness and psychological inflexibility and experiential avoidance-related processes are relatively stable psychological characteristics, short-term physical exercise may not adequately capture their associations with these variables. Therefore, the recall period for physical exercise was extended from the 1-month period commonly used in the original scale to 3 months. This modification was intended to better reflect relatively stable patterns of physical exercise participation, but it may also affect the direct comparability of the present findings with previous studies using the PARS-3. The relevant results should therefore be interpreted with caution. In the present study, Cronbach’s α for this scale was 0.856.

#### 2.2.2 Mindfulness

Mindfulness was measured using the Mindful Attention Awareness Scale (MAAS). The scale was developed by Brown and Ryan (2003) and later revised in Chinese by Chen et al. (2012) It assesses trait mindfulness in daily life. The MAAS is a unidimensional scale comprising 15 items rated on a 6-point scale, with higher scores indicating higher levels of trait mindfulness.

In this study, mindfulness refers to relatively stable trait or dispositional mindfulness, rather than formal mindfulness practice or momentary mindful states during physical exercise. Cronbach’s α for this scale was 0.867.

#### 2.2.3 Psychological inflexibility and experiential avoidance-related scores

Psychological inflexibility and experiential avoidance-related processes were measured using the Acceptance and Action Questionnaire-II (AAQ-II). The AAQ-II was further developed and refined by Fledderus et al. (2012) and its Chinese version was revised for use among college students by Cao et al. (2013). The scale consists of seven items rated on a 7-point scale. Higher scores indicate greater psychological inflexibility and stronger experiential avoidance-related tendencies.

Because the AAQ-II has often been discussed in prior research as a measure of psychological inflexibility and experiential avoidance-related processes, rather than as a pure and specific measure of experiential avoidance alone, this variable is described cautiously as “psychological inflexibility and experiential avoidance-related scores.” Cronbach’s α for this scale was 0.900. The dataset used for the present reanalysis contained only scale total scores and did not include item-level responses; therefore, internal consistency, McDonald’s ω, and the factor structure of the AAQ-II could not be recalculated in the current sample. This issue is further addressed in the limitations section.

### 2.2.4 Depressive symptoms

Depressive symptoms were measured using the Self-Rating Depression Scale (SDS). The scale was developed by Zung (1965) and consists of 20 items rated on a 4-point scale. It assesses the self-reported severity of depressive symptoms over the past week. The SDS raw total score is obtained by summing the 20 items. In the present study, the SDS raw score was used as a continuous variable in the correlation analyses, regression path models, and serial indirect association analyses.

For descriptive classification, the SDS standard score was calculated as follows: SDS standard score = floor (raw score × 1.25). According to commonly used Chinese criteria, an SDS standard score < 53 indicates no depressive symptoms, 53–62 indicates mild depressive symptoms, 63–72 indicates moderate depressive symptoms, and > 72 indicates severe depressive symptoms. In this study, “depression” or “depressive symptoms” refers to the severity of self-reported depressive symptoms measured by the SDS, rather than a clinical diagnosis of depressive disorder. Cronbach’s α for this scale was 0.875.

### 2.3 Data screening and variable construction

Before statistical analysis, the dataset was systematically checked for missing values, duplicated IDs, fully duplicated records, duplicate records based on the core study variables, out-of-range values, and potential outliers. The final analytic dataset comprised 308 observations, with no missing values, duplicated IDs, fully duplicated records, duplicate records on the core study variables, or values outside the theoretical range of each variable. Potential outliers were identified using z-scores > 3.29 and the interquartile range (IQR) method. Although some observations were flagged as potential outliers, all values fell within the theoretical range of the corresponding scales, and there was no evidence that they reflected data entry errors or invalid responses. Therefore, all valid observations were retained for subsequent analyses.

According to the study aims and statistical model requirements, the following variables were further constructed: Gender, SDS_standard, Depression_level, and Exercise_level. In the original sex variable, 1 indicated female and 2 indicated male. For the regression and serial indirect association analyses, sex was recoded as 0 = female and 1 = male. The Self-Rating Depression Scale (SDS) standard score was used for descriptive classification of depressive symptoms, and Exercise_level was used to describe physical exercise volume.

### 2.4 Statistical analysis

All statistical analyses were conducted using R. Descriptive statistics were used to summarize the sample characteristics and main study variables. Because the sex distribution was unbalanced and some variables may have had unequal variances across sex groups, sex differences were examined using Welch’s *t*-test, with Hedges’ g reported as the effect size. Pearson correlation analyses were used to examine bivariate associations among physical exercise, depressive symptoms, trait mindfulness, and Acceptance and Action Questionnaire-II (AAQ-II)-related scores.

Sex was included as a covariate in the regression path models and serial indirect association analyses. Continuous variables were standardized before analysis to allow standardized regression coefficients to be reported and to improve comparability across path estimates. Based on the theoretical model, four regression path models were estimated: first, Depression ∼ Gender + Exercise; second, Mindfulness ∼ Gender + Exercise; third, AAQ-II / experiential avoidance-related scores ∼ Gender + Exercise + Mindfulness; and fourth, Depression ∼ Gender + Exercise + Mindfulness + AAQ-II / experiential avoidance-related scores.

A serial model equivalent to PROCESS Model 6 (Hayes, 2017) was used to examine cross-sectional indirect associations. The model was specified as Exercise → Mindfulness → AAQ-II / experiential avoidance-related scores → Depressive symptoms. Bootstrap resampling was performed with 5,000 resamples. An indirect association was considered statistically significant when the 95% bootstrap confidence interval did not include zero. Because all variables were measured at a single time point, the regression paths and bootstrap estimates were interpreted as cross-sectional associations or cross-sectional indirect associations, rather than causal mediation effects. Thus, the analysis examined whether the data were consistent with the theoretically specified serial association pattern, but it could not establish temporal ordering or causal relations among physical exercise, mindfulness, psychological inflexibility and experiential avoidance-related processes, and depressive symptoms.

To assess the robustness of the regression models, additional diagnostics were conducted, including the variance inflation factor (VIF), tolerance, Cook’s distance, the Breusch–Pagan test, the Shapiro–Wilk test, and residual diagnostics. When evidence of heteroscedasticity was observed, heteroscedasticity-consistent type 3 (HC3) robust standard errors were used in sensitivity analyses to evaluate whether the main path estimates remained stable. Because class identifiers were not retained in the analytic dataset, intraclass correlation coefficients (ICCs) could not be estimated, and cluster-robust standard errors or multilevel models could not be used to account for the nesting of students within physical education classes. In addition, potential confounders such as age, grade, sleep, academic stress, socioeconomic status, and previous mental health status were not collected; therefore, additional covariate-adjusted analyses could not be conducted. These issues are further addressed in the limitations section.

## 3 Results

### 3.1 Data screening, sample characteristics, and depressive symptom distribution

The final analytic dataset included 308 valid questionnaires from 320 invited students, yielding a valid response rate of 96.25%. Data screening showed no missing values, duplicated IDs, fully duplicated records, or duplicate records based on the core study variables. All variable values fell within their theoretical ranges. After potential outliers were screened using z-scores > 3.29 and the interquartile range method, several observations were flagged as potential outliers; however, none exceeded the theoretical range of the corresponding scale, and all were retained for subsequent analyses (Supplementary Table 1).

Among the 308 participants, 198 were female (64.29%) and 110 were male (35.71%). Regarding physical exercise volume, 191 students (62.01%) were classified as having low exercise volume, 93 (30.19%) as having moderate exercise volume, and 24 (7.79%) as having high exercise volume (Table 1). Based on Self-Rating Depression Scale (SDS) standard scores, 244 students (79.22%) showed no depressive symptoms, 54 (17.53%) showed mild depressive symptoms, 8 (2.60%) showed moderate depressive symptoms, and 2 (0.65%) showed severe depressive symptoms. Overall, 64 students met the threshold for at least mild depressive symptoms, corresponding to a prevalence of 20.78%.

**TABLE 1:** Sample characteristics and depressive symptom distribution.

Characteristic	n	Percentage
Total sample size	308	96.25%
Valid response rate	308/320	
Gender		
Female	198	64.29%
Male	110	35.71%
Exercise volume		
Low exercise volume	191	62.01%
Moderate exercise volume	93	30.19%
High exercise volume	24	7.79%
Depressive symptom distribution (SDS standard score)		
No depression	244	79.22%
Mild depressive symptoms	54	17.53%
Moderate depressive symptoms	8	2.60%
Severe depressive symptoms	2	0.65%
Overall prevalence of depressive symptoms	64	20.78%

### 3.2 Gender differences in study variables

Given the unbalanced gender distribution, Welch’s *t*-test was used to examine gender differences in the main study variables. Male students had significantly higher physical exercise scores than female students (female: 16.82 ± 11.57; male: 23.36 ± 17.27; *t* = −3.55, df = 164.47, *p* < 0.001, Hedges’ g = −0.470). Male students also had significantly higher Acceptance and Action Questionnaire-II (AAQ-II)-related scores than female students (female: 19.02 ± 6.95; male: 21.20 ± 7.15; *t* = −2.59, df = 219.96, *p* = 0.010, Hedges’ g = −0.310; Table 2).

**TABLE 2:** Descriptive statistics and gender differences in study variables.

Variable	Female (*n* = 198) M ± SD	Male (*n* = 110) M ± SD	Overall (*N* = 308) M ± SD	*t*	df	*p*	Hedges’ g
Exercise	16.82 ± 11.57	23.36 ± 17.27	19.16 ± 14.20	−3.55	164.47	< 0.001***	−0.470
SDS raw score	36.47 ± 6.76	37.61 ± 8.14	36.88 ± 7.29	−1.25	192.77	0.214	−0.156
Mindfulness	59.01 ± 10.55	59.25 ± 10.66	59.10 ± 10.57	−0.19	223.33	0.847	−0.023
AAQ-II	19.02 ± 6.95	21.20 ± 7.15	19.80 ± 7.09	−2.59	219.96	0.010**	−0.310

Values are presented as M ± SD. Welch’s *t*-tests were used for gender comparisons. Group was coded as 1 = female and 2 = male.

No significant gender difference was observed in SDS raw scores (female: 36.47 ± 6.76; male: 37.61 ± 8.14; *t* = −1.25, df = 192.77, *p* = 0.214). Mindfulness scores also did not differ significantly by gender (female: 59.01 ± 10.55; male: 59.25 ± 10.66; *t* = −0.19, df = 223.33, *p* = 0.847). Because gender differences were observed in physical exercise and AAQ-II-related scores, gender was included as a covariate in the subsequent regression path models and bootstrap indirect association analyses.

### 3.3 Bivariate correlations among physical exercise, depressive symptoms, mindfulness, and AAQ-II scores

Pearson correlation analyses showed that physical exercise was significantly negatively correlated with depressive symptoms (*r* = −0.235, *p* < 0.001), significantly positively correlated with mindfulness (*r* = 0.275, *p* < 0.001), and significantly negatively correlated with AAQ-II-related scores (*r* = −0.176, *p* < 0.01). Depressive symptoms were significantly negatively correlated with mindfulness (*r* = −0.421, *p* < 0.001) and significantly positively correlated with AAQ-II-related scores (*r* = 0.603, *p* < 0.001). Mindfulness was significantly negatively correlated with AAQ-II-related scores (*r* = −0.496, *p* < 0.001; Table 3). These findings provided preliminary statistical support for testing the theoretically specified cross-sectional serial indirect association model.

**TABLE 3:** Pearson correlations among physical exercise, depressive symptoms, mindfulness, and experiential avoidance-related scores.

Variable	M	SD	1	2	3	4
1. Physical exercise	19.16	14.20	–	–	–	–
2. Depressive symptoms	36.88	7.29	−0.235***			
3. Mindfulness	59.10	10.57	0.275***	−0.421***		
4. Experiential avoidance-related scores	19.80	7.09	−0.176**	0.603***	−0.496***	

*N* = 308. *p < 0.05, **p < 0.01, ***p < 0.001.

### 3.4 Regression path models for the cross-sectional serial mediation model

Regression path models were estimated to examine the theoretically specified cross-sectional serial model. All continuous variables were standardized, and gender was coded as 0 = female and 1 = male. In the total association model, physical exercise was significantly negatively associated with depressive symptoms [β = −0.264, standard error (SE) = 0.057, t = −4.67, *p* < 0.001, 95% confidence interval (CI) (−0.375, −0.153)]. Physical exercise was also significantly positively associated with mindfulness [β = 0.286, SE = 0.056, *t* = 5.08, *p* < 0.001, 95% CI (0.176, 0.397); Table 2, 4)].

**TABLE 4:** Regression path models for the cross-sectional serial mediation analysis.

Dependent variable	Predictor	*R* ^2^	*F*	Model p	β	SE	*T*	*P*	95% CI	
									Lower	Upper
Depression	Gender Exercise	0.072	11.82	< 0.001	0.133	0.118	2.36	0.019*	−0.098	0.364
					−0.264	0.057	−4.67	< 0.001***	−0.375	−0.153
Mindfulness	Gender Exercise	0.078	12.91	< 0.001	−0.052	0.117	−0.93	0.355	−0.282	0.178
					0.286	0.056	5.08	< 0.001***	0.176	0.397
AAQ	Gender Exercise Mindfulness	0.275	38.45	< 0.001	0.171	0.104	3.41	< 0.001***	−0.034	0.376
					−0.083	0.052	−1.59	0.113	−0.185	0.019
					−0.475	0.051	−9.33	< 0.001***	−0.574	−0.375
Depression	Gender Exercise Mindfulness AAQ	0.395	49.42	< 0.001	0.026	0.097	0.56	0.579	−0.165	0.217
					−0.113	0.048	−2.35	0.019*	−0.207	−0.019
					−0.137	0.053	−2.60	0.010**	−0.241	−0.034
					0.512	0.052	9.75	< 0.001***	0.409	0.615

*N* = 308. All continuous variables were standardized. Gender was coded as 0 = female, 1 = male. β = standardized regression coefficient. CI, confidence interval.

In the model with AAQ-II-related scores as the outcome, the direct association between physical exercise and AAQ-II-related scores was not significant after mindfulness was included [β = −0.083, SE = 0.052, *t* = −1.59, *p* = 0.113, 95% CI (−0.185, 0.019)]. In contrast, mindfulness was significantly negatively associated with AAQ-II-related scores [β = −0.475, SE = 0.051, *t* = −9.33, *p* < 0.001, 95% CI (−0.574, −0.375)]. This finding indicated that physical exercise did not show an independent direct statistical association with AAQ-II-related scores after mindfulness was considered. In the final model, mindfulness was significantly negatively associated with depressive symptoms [β = −0.137, SE = 0.053, *t* = −2.60, *p* = 0.010, 95% CI (−0.241, −0.034)], whereas AAQ-II-related scores were significantly positively associated with depressive symptoms [β = 0.512, SE = 0.052, *t* = 9.75, *p* < 0.001, 95% CI (0.409, 0.615)]. After controlling for both mindfulness and AAQ-II-related scores, the direct association between physical exercise and depressive symptoms remained significant [β = −0.113, SE = 0.048, *t* = −2.35, *p* = 0.019, 95% CI (−0.207, −0.019)].

### 3.5 Bootstrap analysis of cross-sectional indirect associations

**FIGURE 1:**
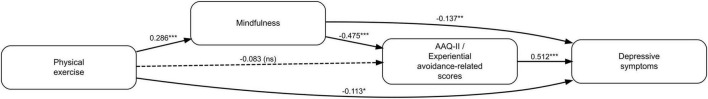
Path diagram of the serial mediation model.

Bootstrap resampling with 5,000 resamples was used to further examine the cross-sectional indirect associations. Because this study used a cross-sectional design, the following paths were interpreted as cross-sectional indirect associations rather than causal mediation effects. The indirect association between physical exercise and depressive symptoms through mindfulness was significant [estimate = −0.0392, bootstrap 95% CI (−0.0862, −0.0091)], accounting for 14.9% of the total association. The indirect association between physical exercise and depressive symptoms through Acceptance and Action Questionnaire-II (AAQ-II)-related scores was not significant [estimate = −0.0424, bootstrap 95% CI (−0.1044, 0.0214)], accounting for 16.1% of the total association. The cross-sectional serial indirect association between physical exercise and depressive symptoms through mindfulness and AAQ-II-related scores was significant [estimate = −0.0695, bootstrap 95% CI (−0.1096, −0.0420)], accounting for 26.3% of the total association (Figure 1 and Table 5).

**TABLE 5:** Bootstrap analysis of standardized indirect associations in the cross-sectional serial mediation model.

Path type	Mediation path	Estimate	Boot LLCI	Boot ULCI	% Of total	Significance
Indirect path 1	Exercise → mindfulness → depression	−0.0392	−0.0862	−0.0091	14.9%	Significant
Indirect path 2	Exercise → AAQ → depression	−0.0424	−0.1044	0.0214	16.1%	Not significant
Indirect path 3	Exercise → mindfulness → AAQ → depression	−0.0695	−0.1096	−0.0420	26.3%	Significant
Total indirect association	Sum of all indirect paths	−0.1512	−0.2250	−0.0831	57.3%	Significant
Direct association	Exercise → depression	−0.1128	−0.2194	−0.0122	42.7%	Significant

*N* = 308. Estimates are standardized. Bootstrap confidence intervals were based on 5,000 resamples. Because the data are cross-sectional, the indirect paths should be interpreted as cross-sectional indirect associations rather than causal mediation effects. Gender (0 = female, 1 = male) was included as a covariate.

The total indirect association was significant [estimate = −0.1512, bootstrap 95% CI (−0.2250, −0.0831)], accounting for 57.3% of the total association. The direct association between physical exercise and depressive symptoms remained significant [estimate = −0.1128, bootstrap 95% CI (−0.2194, −0.0122)], accounting for 42.7% of the total association. The total association was also significant [estimate = −0.2640, bootstrap 95% CI (−0.3744, −0.1524)]. Accordingly, Hypothesis 1 was supported, indicating a significant indirect association between physical exercise and depressive symptoms through mindfulness. Hypothesis 2 was not supported, as the indirect association through AAQ-II-related scores alone was not statistically significant. Hypothesis 3 was supported, indicating a significant cross-sectional serial indirect association through mindfulness and AAQ-II-related scores.

### 3.6 Measurement checks, common method variance, regression diagnostics, and sensitivity analyses

Internal consistency, McDonald’s ω, and factor structure were not recalculated from item-level data in this study. Given that the AAQ-II is commonly interpreted as reflecting psychological inflexibility and experiential avoidance-related processes, AAQ-II scores were interpreted cautiously and were not treated as a pure measure of experiential avoidance (Supplementary Table 2). Regarding common method variance, the previously reported Harman single-factor test identified 12 factors with eigenvalues > 1, and the first factor explained 29.21% of the variance (Podsakoff et al., 2003). This result did not indicate that a single factor dominated the overall covariance structure. However, because all main variables were assessed using self-report scales at the same time point, common method variance could not be completely ruled out (Supplementary Table 3).

**FIGURE 2:**
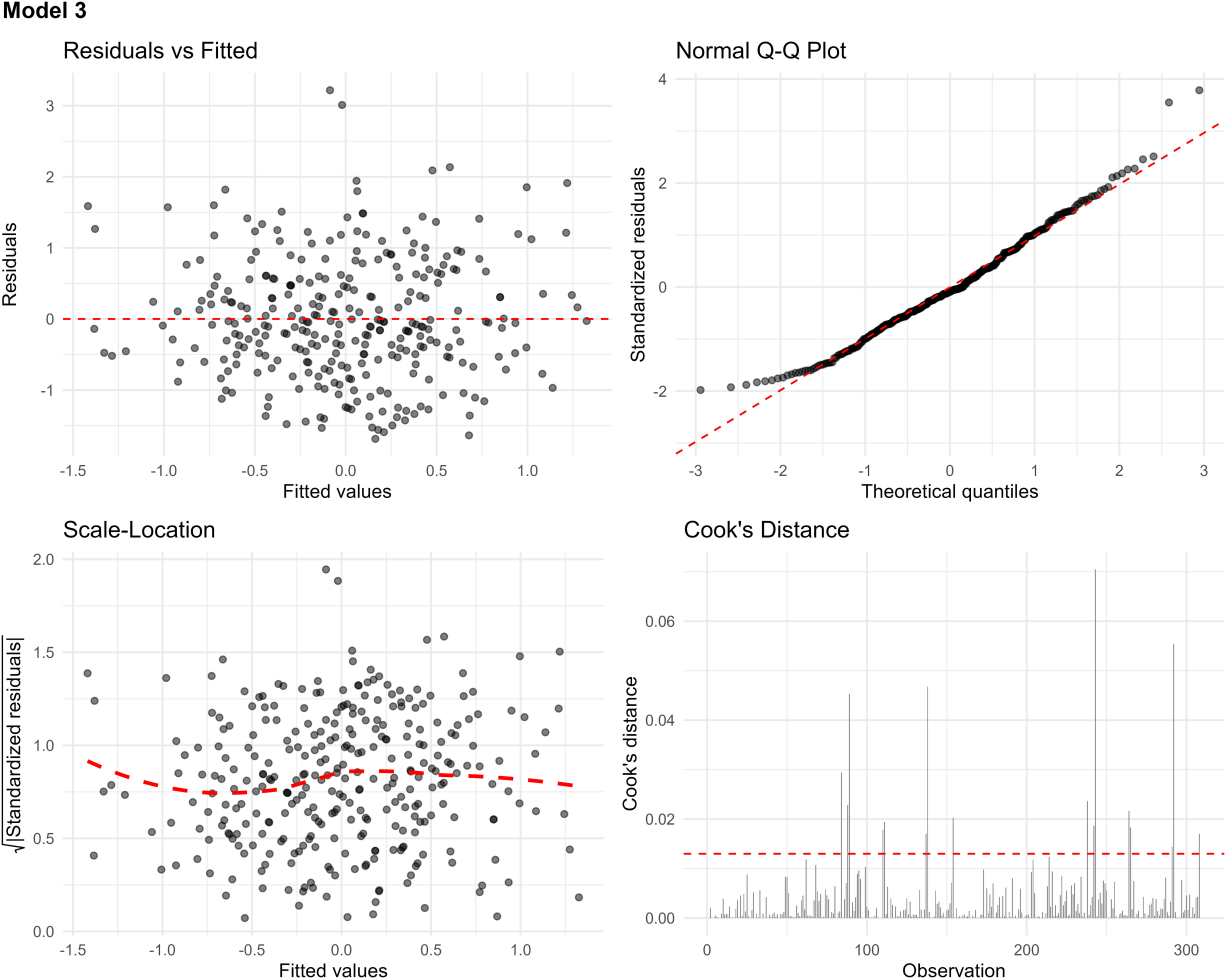
Regression diagnostic plots for Model 3.

Regression diagnostics showed that all variance inflation factor (VIF) values were below 5, with VIFs ranging from 1.051 to 1.395, indicating no serious multicollinearity. Cook’s distance identified several potentially influential observations, whose influence on the main results was considered together with the sensitivity analyses. The Breusch–Pagan tests indicated evidence of heteroscedasticity in Model 3 and Model 4 (BP = 12.803, *p* = 0.005; BP = 18.456, *p* = 0.001, respectively). Therefore, sensitivity analyses were conducted using heteroscedasticity-consistent type 3 (HC3) robust standard errors (Supplementary Table 4 and Figure 2).

The HC3 robust standard error sensitivity analyses showed that the main path conclusions remained unchanged. The direct association between physical exercise and AAQ-II-related scores remained nonsignificant (HC3 *p* = 0.181). The total association between physical exercise and depressive symptoms remained significant (HC3 *p* < 0.001), as did the association between physical exercise and mindfulness (HC3 *p* < 0.001), mindfulness and AAQ-II-related scores (HC3 *p* < 0.001), mindfulness and depressive symptoms (HC3 *p* = 0.017), and AAQ-II-related scores and depressive symptoms (HC3 *p* < 0.001). The direct association between physical exercise and depressive symptoms also remained significant under robust standard errors (HC3 *p* = 0.040; Supplementary Table 5). Additional confounders, including age, body mass index (BMI), sleep quality, academic stress, socioeconomic status, and previous mental health status, were not included in the study; therefore, further covariate-adjusted analyses could not be conducted.

## 4 Discussion

From the perspective of Acceptance and Commitment Therapy (ACT), this study examined the cross-sectional associations among physical exercise, trait mindfulness, psychological inflexibility and experiential avoidance-related processes, and depressive symptoms in college students. The revised analyses showed that physical exercise was significantly negatively associated with depressive symptoms, and that this association was statistically linked to mindfulness and to AAQ-II-related scores following mindfulness. Notably, AAQ-II-related scores did not constitute an independent indirect pathway, but operated primarily within the serial pathway of physical exercise, mindfulness, AAQ-II-related scores, and depressive symptoms. Therefore, the present findings should not be interpreted as evidence of causal mediation, but rather as a pattern of cross-sectional indirect associations consistent with ACT.

### 4.1 Depressive symptom levels among college students

SDS standard scores, the prevalence of at least mild depressive symptoms in this sample was 20.78%. This estimate was lower than those reported in some previous studies of depressive symptoms among college students. For example, a prior systematic review reported a global prevalence of depressive symptoms among college students of approximately 33.6% (Li et al., 2022), while the prevalence among Chinese college students was reported to be approximately 34.70% (Lin et al., 2025). The discrepancy between the present findings and previous estimates may be related to differences in sample source, measurement instrument, classification criteria, survey period, and the recruitment of participants from general physical education classes. Because the present sample was drawn from a single university in Jiangsu Province and depressive symptoms were screened using the SDS self-report scale, the observed prevalence should not be directly generalized to the broader population of Chinese college students.

The gender difference analysis showed no significant difference in depressive symptom scores between male and female students. This finding suggests that mental health screening and intervention among college students should not simply assume that one gender group is at higher risk; rather, assessment should consider the characteristics of the specific sample and relevant psychosocial and behavioral factors. At the same time, male students reported significantly higher physical exercise scores and AAQ-II-related scores than female students. Accordingly, gender was included as a covariate in the subsequent regression path models and bootstrap indirect association analyses. It should be emphasized that controlling for gender can only partially reduce the influence of the unbalanced gender distribution on statistical estimates and cannot eliminate the external validity limitations arising from limited sample representativeness.

### 4.2 Association between physical exercise and depressive symptoms

The findings indicated that physical exercise was significantly negatively associated with depressive symptoms. In other words, students with higher levels of physical exercise tended to report lower levels of depressive symptoms in this sample. This result is broadly consistent with previous research on physical activity and mental health among college students. Xiang et al. (2020) found that participation in physical exercise was associated with lower levels of negative emotions, including anxiety and depression, among college students. Prior studies have proposed several biological and psychosocial explanations for the association between physical exercise and depressive symptoms. Hird et al. (2024) suggested that exercise may be related to biological processes involving inflammation, reward systems, dopamine, and serotonergic function, whereas Chang (2025) emphasized the roles of self-efficacy and social support in the association between physical activity and depressive symptoms.

Most participants in the present study had low exercise volume. Therefore, the current findings only indicate a cross-sectional negative association between exercise volume and depressive symptoms; they do not support the inference that low-dose physical exercise is sufficient to improve mental health, nor can they identify the optimal type, intensity, frequency, or duration of exercise. Existing evidence has not reached a consensus regarding the relationship between exercise dose and depressive outcomes. Longitudinal evidence also indicates that the association between physical activity and depression may differ across activity domains, suggesting that total exercise volume alone may not fully capture the complexity of this relationship (Werneck et al., 2023). Herring et al. (2010) reported that higher exercise levels were associated with a lower risk of depression, whereas other studies have suggested that the psychological effects of different exercise intensities may vary (Zeng and Wang, 2025). Such differences may be attributable to variations in participant age, living environment, sources of stress, exercise type, and measurement approaches. Joshi et al. (2016) also indicated that different types of physical activity may be differentially associated with depressive outcomes. Similarly, domain-specific analyses in a large Brazilian adult sample found that different physical activity domains were independently associated with depressive symptoms, further supporting the need to avoid treating physical activity as a homogeneous exposure (Werneck et al., 2020). Accordingly, the present study does not draw practical conclusions about exercise dose, but instead treats physical exercise as a behavioral factor associated with depressive symptoms, providing preliminary evidence for future longitudinal and intervention studies.

### 4.3 Indirect association through mindfulness between physical exercise and depressive symptoms

The bootstrap analysis showed a significant indirect association between physical exercise and depressive symptoms through mindfulness. This finding suggests that trait mindfulness may be an important psychological factor for understanding the association between physical exercise and depressive symptoms among college students. Previous research has also suggested that mindfulness may play an important role in the association between physical activity and depressive symptoms in college students (Zhao et al., 2024). The present study used the Mindful Attention Awareness Scale (MAAS) to measure trait mindfulness, rather than formal mindfulness training or momentary mindful states during exercise. Therefore, the findings should be interpreted as reflecting an association between physical exercise level and individualsindividualstion between physical exercndfulness traininas evidence that physical exercise directly produces mindfulness practice effects.

From an emotion regulation perspective, mindfulness is closely related to how individuals are aware of, accept, and regulate emotional experiences. Roemer et al. (2015) identified mindfulness as an important protective factor in emotion regulation. According to Grossemot1998) model of emotion regulation, individualsd regulate, appraisal, and responses to emotional experiences influence the duration and expression of emotions. Wang et al. (2025) further suggested that mindfulness may be associated with lower emotional distress through processes such as decentering. Bodily awareness, attentional engagement, and action-oriented participation during physical exercise may share some features with the present-moment awareness emphasized in mindfulness. Cavour-Wiêcławek and Rogowska (2024) also noted links between attentional styles in physical activity and yoga practice and mindfulness-related characteristics. In this context, the present findings may be understood as indicating that students with higher levels of physical exercise tended to report higher trait mindfulness, which in turn was associated with lower depressive symptoms.

### 4.4 Serial indirect association through mindfulness and AAQ-II-related scores

The finding that requires the most cautious interpretation is that the direct association between physical exercise and AAQ-II-related scores was not significant after mindfulness was included, and the indirect association from physical exercise to depressive symptoms through AAQ-II-related scores alone was also nonsignificant. Therefore, the present findings do not support interpreting AAQ-II-related scores as an independent mediator between physical exercise and depressive symptoms. Instead, AAQ-II-related scores appeared to operate mainly within the serial pathway following mindfulness. In other words, the data support a serial rather than parallel interpretation: physical exercise was associated with higher trait mindfulness, higher trait mindfulness was further associated with lower AAQ-II-related scores, and lower AAQ-II-related scores were associated with lower depressive symptoms.

This finding helps clarify the theoretical relationship between mindfulness and experiential avoidance-related processes. Experiential avoidance is commonly regarded as an important manifestation of psychological inflexibility and is closely linked to depressive symptoms (Mellick et al., 2017). Individuals with higher psychological inflexibility may be more likely to avoid or suppress unwanted internal experiences, a coping pattern that may be associated with more severe psychological problems (Na et al., 2022; Shen et al., 2024). Ford et al. (2018) also suggested that acceptance of negative emotions and thoughts is closely related to psychological health. By contrast, mindfulness emphasizes awareness, openness, and nonjudgmental attention to present experience, which may help individuals relate to internal experiences with less avoidance and resistance. Schut and Boelen (2017) likewise noted that mindfulness, rumination, and experiential avoidance are all closely associated with depressive symptoms.

The nonsignificant pathway through AAQ-II-related scores alone does not mean that psychological inflexibility and experiential avoidance-related processes are unimportant in the model. Rather, it suggests that their role may depend on the more upstream psychological process of mindfulness. In the present study, physical exercise was not directly associated with AAQ-II-related scores after mindfulness was controlled. Instead, the statistical link between physical exercise and AAQ-II-related scores was primarily characterized by mindfulness. Thus, AAQ-II-related scores are better understood as a downstream psychological process following mindfulness, rather than as an independent parallel pathway alongside mindfulness.

It should also be noted that the AAQ-II was used in this study to assess psychological inflexibility and experiential avoidance-related processes, rather than as a pure measure of experiential avoidance. Previous studies have suggested that physical exercise may moderate the relationship between experiential avoidance and depression (Olmedilla et al., 2010), and that enjoyable group activities may be associated with lower avoidance tendencies (Hayes et al., 1996). In the present data, however, AAQ-II-related scores did not show an independent mediating role, but were statistically meaningful within the serial pathway following mindfulness. This finding suggests that future research examining physical exercise, mindfulness, and experiential avoidance-related processes should further distinguish the attentional and acceptance components of mindfulness, as well as different dimensions of psychological inflexibility.

Monitoring and Acceptance Theory (MAT) proposes that mindfulness comprises two core components: monitoring and acceptance, with the acceptance component potentially playing a particularly important role in alleviating negative emotions (Anâlayo, 2022). The present study did not directly measure the monitoring and acceptance components of mindfulness; therefore, the current data cannot determine which component played a stronger role. Nevertheless, the serial pathway linking mindfulness to AAQ-II-related scores suggests that the acceptance-related features of mindfulness may be a valuable direction for future research. Given the cross-sectional design, this interpretation should be regarded as a cautious theoretical inference and does not demonstrate that mindfulness temporally precedes changes in psychological inflexibility or experiential avoidance-related processes.

### 4.5 Theoretical and practical implications

From the perspective of Acceptance and Commitment Therapy (ACT), this study provides preliminary evidence for understanding the association between physical exercise and depressive symptoms among college students. Rather than supporting a model in which mindfulness and experiential avoidance function as two parallel mediators, the results are more consistent with an ordered serial interpretation: physical exercise was associated with trait mindfulness, and trait mindfulness was further associated with psychological inflexibility and experiential avoidance-related processes as well as depressive symptoms. This pattern aligns the theoretical model more closely with the observed data and avoids overinterpreting AAQ-II-related scores as an independent mediator.

At the practical level, the findings suggest that future research on exercise-based mental health promotion among college students may benefit from closer attention to awareness-, attention-, and acceptance-related psychological characteristics during physical activity participation. However, the present study cannot provide direct recommendations regarding exercise dose, nor can it show that physical exercise itself changes mindfulness or psychological inflexibility. A more cautious interpretation is that trait mindfulness and AAQ-II-related scores may be useful psychological indicators to include in future exercise intervention studies. Longitudinal or randomized controlled studies could further examine whether integrating mindfulness training, acceptance components, or values-based action into physical exercise programs is associated with changes in depressive symptoms.

## 5 Conclusion

From the perspective of ACT, this study examined the cross-sectional associations among physical exercise, trait mindfulness, psychological inflexibility and experiential avoidance-related processes, and depressive symptoms in college students. The findings showed that physical exercise was associated with lower depressive symptoms, and that this association was partly characterized by cross-sectional indirect associations through mindfulness and through AAQ-II-related scores following mindfulness. AAQ-II-related scores did not constitute an independent indirect pathway, but operated mainly within the serial pathway following mindfulness. Because this study used a cross-sectional design, the findings should not be interpreted as evidence of causal mediation. Future studies should use longitudinal, ecological momentary assessment, or randomized intervention designs to further examine the temporal ordering and potential causal relationships among physical exercise, mindfulness, psychological inflexibility and experiential avoidance-related processes, and depressive symptoms.

## 6 Limitations and future directions

This study has several limitations:

(1) The sample was recruited from a single university in Jiangsu Province, China, and included a relatively high proportion of female participants. Although gender was controlled for in the regression path models and indirect association analyses, this adjustment cannot fully address the limitations in external validity arising from the single-site sample and gender imbalance. Therefore, the findings should be generalized with caution. Future studies should use multi-region, multi-university samples with more balanced gender distributions to further validate these findings.
(2) This study used a cross-sectional design, with all variables measured at the same time point. As a result, the temporal ordering among physical exercise, mindfulness, AAQ-II-related scores, and depressive symptoms could not be determined, and reverse or bidirectional associations cannot be ruled out. The indirect paths identified in this study should therefore be interpreted as cross-sectional indirect associations rather than causal mediation effects. Future research should use longitudinal follow-up, ecological momentary assessment, or randomized controlled designs to further examine the temporal relationships among these variables.
(3) Participants were recruited from 11 general physical education classes, but class identifiers were not retained in the analytic dataset. Therefore, intraclass correlation coefficients could not be estimated, and cluster-robust standard errors or multilevel models could not be used to account for the nested class structure. In addition, only gender was controlled for in this study; potential confounders such as age, grade, sleep quality, academic stress, socioeconomic status, and previous mental health status were not collected. Future studies should retain class-level information and include a broader range of individual and contextual variables.
(4) Only total scores were available for the AAQ-II, which prevented re-examination of its factor structure, McDonald’s omega (ω), or item-level reliability. Given that the AAQ-II is more appropriately interpreted as an indicator of psychological inflexibility and experiential avoidance-related processes, future research should use item-level data, confirmatory factor analysis, and additional acceptance-related measures to further evaluate its applicability among Chinese college students.
(5) All main variables were assessed using self-report scales at a single time point. Although Harmanlege stud-factor test did not indicate that a single factor dominated the overall covariance structure, common method variance cannot be completely ruled out. In addition, the recall period of the Physical Activity Rating Scale-3 (PARS-3) was extended from 1 to 3 months, which may affect comparability with previous studies. Future research should incorporate objective physical activity monitoring, multi-source psychological assessment, and comparisons across different recall periods to improve measurement stability and the accuracy of interpretation.

## Data Availability

The raw data supporting the conclusions of this article will be made available by the authors, without undue reservation.
