# Supplementary material for: Physical exercise and depression in college students: the serial mediating roles of mindfulness and experiential avoidance
Source: Front Psychol. 2026 Jun 22;17:1838963. doi: 10.3389/fpsyg.2026.1838963 (PMC13334697; doi:10.3389/fpsyg.2026.1838963)
Supplement: Supplementary file 1 [file Table_1.docx]

| Supplementary Table S1. Data screening and quality checks | | | |
| --- | --- | --- | --- |
| Section | Item | Value | Details |
| A. Sample Size and Response Rate | Initial invited participants | 320 |  |
|  | Valid responses (actual N) | 308 |  |
|  | Valid response rate | 96.25% | N / 320 * 100 |
| B. Missing Data | ID | 0 | n missing |
|  | Exercise | 0 | n missing |
|  | Mindfulness | 0 | n missing |
|  | Depression | 0 | n missing |
|  | Group | 0 | n missing |
|  | AAQ | 0 | n missing |
| C. Duplicate Checks | Duplicated IDs | 0 | n |
|  | Fully duplicated rows | 0 | n |
|  | Duplicates on core variables | 0 | Exercise, Mindfulness, Depression, Group, AAQ |
| D. Variable Range Checks | Exercise | 0 out of range | Observed: [0, 80], expected: [0, 100] |
|  | Mindfulness | 0 out of range | Observed: [36, 88] |
|  | Depression (SDS raw) | 0 out of range | Observed: [20.0, 64.0], expected: [0, 80] |
|  | Group | 0 out of range | Values: 1, 2, expected: 1 or 2 |
|  | AAQ-II | 0 out of range | Observed: [7, 42], expected: [0, 84] |
| E. Potential Outliers | Exercise | z: 2; IQR: 7 | z-score > 3.29 or 1.5*IQR rule |
|  | Mindfulness | z: 0; IQR: 2 | z-score > 3.29 or 1.5*IQR rule |
|  | Depression | z: 2; IQR: 2 | z-score > 3.29 or 1.5*IQR rule |
|  | AAQ-II | z: 0; IQR: 4 | z-score > 3.29 or 1.5*IQR rule |
| F. Derived Variable Checks | Gender | 308 | Valid entries (Female or Male) |
|  | SDS_standard | 308 | Valid entries (0-100 range) |
|  | Depression_level | 308 | Valid depression level categories |
|  | Exercise_level | 308 | Valid exercise level categories |

| Supplementary Table S2. Reliability and validity evidence for the study scales | | | |
| --- | --- | --- | --- |
| Scale | Number of items | Cronbach's alpha | Source |
| AAQ-II | 7 | 0.900 | Reported in the manuscript, not recalculated |
| MAAS | 15 | 0.867 | Reported in the manuscript, not recalculated |
| PARS-3 | 3 | 0.856 | Reported in the manuscript, not recalculated |
| SDS | 20 | 0.875 | Reported in the manuscript, not recalculated |
| Note. N = 308. The current dataset contains only scale total scores; Cronbach's alpha values were reported in the manuscript and not recalculated here. Because AAQ-II has been debated as a measure of psychological inflexibility and experiential avoidance-related processes, AAQ-II scores should be interpreted cautiously as experiential avoidance-related or psychological inflexibility-related scores. | | | |

Limitation: The current dataset contains only scale total scores, so internal consistency and factor structure cannot be recalculated from item-level responses.

| Supplementary Table S3. Common method variance assessment | | | |
| --- | --- | --- | --- |
| Assessment | Item | Value | Source |
| Limitation | Item-level data availability | Not available | The current dataset contains only scale total scores, so Harman's single-factor test and one-factor CFA cannot be recalculated from item-level responses. |
| Reported CMV Assessment | Number of factors with eigenvalue > 1 | 12 | Reported in the manuscript, not recalculated |
|  | Variance explained by the first factor | 29.21% | Reported in the manuscript, not recalculated |
|  | Interpretation | First factor < 50%; one factor does not dominate |  |
| Note. N = 308. The results did not suggest that one factor dominated the covariance structure; however, common method variance cannot be completely ruled out because all main variables were assessed using self-report questionnaires in the same survey session. | | | |

| Supplementary Table S4. Regression diagnostics for the mediation path models | | | |
| --- | --- | --- | --- |
| Model | Diagnostic | Variable | Value |
| Model 1: Depression ~ Gender + Exercise | VIF / Tolerance | Gender | VIF = 1.051, Tolerance = 0.951 |
|  | VIF / Tolerance | Exercise_z | VIF = 1.051, Tolerance = 0.951 |
|  | Max Cook's distance |  | 0.0477 (threshold = 0.0130) |
|  | Influential observations |  | 18 (Cook's d > 4/N) |
|  | Breusch-Pagan test |  | BP = 4.269, p = 0.118 |
|  | Shapiro-Wilk test |  | W = 0.9822, p = < .001 * |
|  | Residual SE |  | 0.9665 |
|  | Multicollinearity |  | No serious multicollinearity (all VIF < 5) |
| Model 2: Mindfulness ~ Gender + Exercise | VIF / Tolerance | Gender | VIF = 1.051, Tolerance = 0.951 |
|  | VIF / Tolerance | Exercise_z | VIF = 1.051, Tolerance = 0.951 |
|  | Max Cook's distance |  | 0.0642 (threshold = 0.0130) |
|  | Influential observations |  | 14 (Cook's d > 4/N) |
|  | Breusch-Pagan test |  | BP = 3.413, p = 0.182 |
|  | Shapiro-Wilk test |  | W = 0.9914, p = 0.071 |
|  | Residual SE |  | 0.9633 |
|  | Multicollinearity |  | No serious multicollinearity (all VIF < 5) |
| Model 3: AAQ ~ Gender + Exercise + Mindfulness | VIF / Tolerance | Gender | VIF = 1.054, Tolerance = 0.948 |
|  | VIF / Tolerance | Exercise_z | VIF = 1.140, Tolerance = 0.877 |
|  | VIF / Tolerance | Mindfulness_z | VIF = 1.085, Tolerance = 0.922 |
|  | Max Cook's distance |  | 0.0705 (threshold = 0.0130) |
|  | Influential observations |  | 16 (Cook's d > 4/N) |
|  | Breusch-Pagan test |  | BP = 12.803, p = 0.005 * |
|  | Shapiro-Wilk test |  | W = 0.9829, p = < .001 * |
|  | Residual SE |  | 0.8556 |
|  | Multicollinearity |  | No serious multicollinearity (all VIF < 5) |
| Model 4: Depression ~ Gender + Exercise + Mindfulness + AAQ | VIF / Tolerance | Gender | VIF = 1.095, Tolerance = 0.913 |
|  | VIF / Tolerance | Exercise_z | VIF = 1.150, Tolerance = 0.870 |
|  | VIF / Tolerance | Mindfulness_z | VIF = 1.395, Tolerance = 0.717 |
|  | VIF / Tolerance | AAQ_z | VIF = 1.379, Tolerance = 0.725 |
|  | Max Cook's distance |  | 0.0799 (threshold = 0.0130) |
|  | Influential observations |  | 20 (Cook's d > 4/N) |
|  | Breusch-Pagan test |  | BP = 18.456, p = 0.001 * |
|  | Shapiro-Wilk test |  | W = 0.9863, p = 0.005 * |
|  | Residual SE |  | 0.7830 |
|  | Multicollinearity |  | No serious multicollinearity (all VIF < 5) |
| Note. N = 308. All continuous variables were standardized. Gender was coded as 0 = female, 1 = male. VIF = variance inflation factor. Cook's distance threshold = 4/N. No serious multicollinearity was indicated if all VIF values are below 5. Breusch-Pagan test was significant for some models; HC3 robust standard errors are provided as a sensitivity check. | | | |

# Supplementary Table S5. Sensitivity analyses for clustering and additional covariates

| Panel A. Data availability for sensitivity analyses | |
| --- | --- |
| Item | Status |
| Class-level identifiers | Not available in the analytic dataset |
| Additional confounders | Not available in the analytic dataset |
| Intraclass correlations | Could not be estimated |
| Cluster-robust standard errors | Could not be estimated |
| Multilevel models | Could not be estimated |
| Additional covariate-adjusted analyses | Could not be estimated |
| Available sensitivity analysis | HC3 robust standard errors computed (see below) |

| Panel B. HC3 robust standard errors sensitivity analysis | | | | | | | | |
| --- | --- | --- | --- | --- | --- | --- | --- | --- |
| Model | Predictor | Beta | OLS SE | OLS p | OLS 95% CI | HC3 SE | HC3 p | HC3 95% CI |
| Model 1: Depression ~ Gender + Exercise | Gender | 0.278 | 0.1179 | 0.019 | [0.047, 0.509] | 0.1224 | 0.024 | [0.038, 0.518] |
|  | Exercise_z | -0.264 | 0.0566 | < .001 | [-0.375, -0.153] | 0.0592 | < .001 | [-0.380, -0.148] |
| Model 2: Mindfulness ~ Gender + Exercise | Gender | -0.109 | 0.1175 | 0.355 | [-0.339, 0.121] | 0.1141 | 0.341 | [-0.332, 0.115] |
|  | Exercise_z | 0.286 | 0.0564 | < .001 | [0.176, 0.397] | 0.0563 | < .001 | [0.176, 0.397] |
| Model 3: AAQ ~ Gender + Exercise + Mindfulness | Gender | 0.357 | 0.1045 | < .001 | [0.152, 0.561] | 0.1033 | < .001 | [0.154, 0.559] |
|  | Exercise_z | -0.083 | 0.0521 | 0.113 | [-0.185, 0.019] | 0.0618 | 0.181 | [-0.204, 0.038] |
|  | Mindfulness_z | -0.475 | 0.0509 | < .001 | [-0.574, -0.375] | 0.0499 | < .001 | [-0.572, -0.377] |
| Model 4: Depression ~ Gender + Exercise + Mindfulness + AAQ | Gender | 0.054 | 0.0974 | 0.579 | [-0.137, 0.245] | 0.1038 | 0.603 | [-0.149, 0.258] |
|  | Exercise_z | -0.113 | 0.0479 | 0.019 | [-0.207, -0.019] | 0.0546 | 0.040 | [-0.220, -0.006] |
|  | Mindfulness_z | -0.137 | 0.0528 | 0.010 | [-0.241, -0.034] | 0.0571 | 0.017 | [-0.249, -0.025] |
|  | AAQ_z | 0.512 | 0.0525 | < .001 | [0.409, 0.615] | 0.0584 | < .001 | [0.397, 0.626] |
| Note. N = 308. All continuous variables were standardized. Gender was coded as 0 = female, 1 = male. OLS = ordinary least squares. HC3 = heteroscedasticity-consistent standard errors (type HC3). CI = confidence interval. Class-level identifiers were not available in the analytic dataset, so intraclass correlations and cluster-robust standard errors could not be estimated. Additional confounders (e.g., age, BMI, sleep quality) were not available, so covariate-adjusted sensitivity analyses could not be conducted. This limitation should be acknowledged in the manuscript. | | | | | | | | |
